# Effect of saliva contamination on cementation of orthodontic brackets using different adhesive systems

**DOI:** 10.4317/jced.53576

**Published:** 2017-07-01

**Authors:** Aliden-Willian Robaski, Saulo Pamato, Marcelo Tomás-de Oliveira, Jefferson-Ricardo Pereira

**Affiliations:** 1 DDS, MS, Department of Health Science, University of Southern Santa Catarina, Tubarão, SC, Brazil; 2 DDS, PhD, Department of Dental Materials, University of Southern Santa Catarina, Tubarão, SC, Brazil; 3 DDS, PhD, Department of Prosthodontics, University of Southern Santa Catarina, Tubarão, SC, Brazil

## Abstract

**Background:**

The enamel condition and the quality of surface are points that need to be considered for achieving optimal efficiency in the treatment with orthodontic brackets. The aim of this study was to assess the immediate bond strength of metallic brackets cemented to dental.

**Material and Methods:**

Forty human premolars were double-sectioned, placed in PVC matrices and randomly divided into 10 groups (n=8). They received artificial saliva contamination before or after the application of adhesive systems, except for the control groups. The metallic brackets were cemented using two orthodontic cements (Transbond™ Plus Color Change, 3M Unitek e Transbond™ XT Light, 3M Unitek). The specimens were subjected to mechanical shear bond strength testing and classified according to the fracture pattern. The results were analyzed using a two-way ANOVA and Tukey’s test for multiple comparisons (*p*<0.05).

**Results:**

ANOVA analysis showed statistically significant differences between the groups (*p*=0.01). The Tukey’s multiple comparison test indicated statistically significant difference between G6 and G7 groups (*p*<0.05). A high prevalence of adhesive failure in the groups receiving the hydrophobic adhesive system.

**Conclusions:**

The saliva contamination prior to the application of a hydrophobic simplified conventional adhesive system was responsible for decreasing the immediate bond strength values of brackets cemented on the dental enamel.

** Key words:**Bonding, orthodontic brackets, shear bond strength, saliva, adhesive systems.

## Introduction

Orthodontics has shown considerable advancement in recent decades. In addition to the patients’ quest for cosmetic procedures, the use of orthodontic appliances has grown considerably, making it even more important to successfully cementing brackets, which are critical during the course of treatment (1).

The beginning of orthodontic practice has been marked by the use of multi-bandaging technique of teeth; however, this technique fell into disuse because it caused great injury to the gingival tissue, as well as aesthetic concerns. In order to overcome such disadvantages, the cementing technique of brackets directly on the teeth was developed, thus favoring cleansing and allowing for the reduction of treatment cost and time (2).

Orthodontic procedures have become dependent on an effective adhesive system, able to withstand stress and shear bond strength upon the device, and consequently, transmitted to the dental arch (1).

The literature is unanimous in stating that loosening or detachment of orthodontic brackets is caused by failures in the cementing method, because of little retentiveness of certain bracket bases or by the action of masticatory forces. These failures can undermine treatment, delaying the expected results and reducing patient satisfaction (3). Therefore, the enamel condition, the cleansing of the surface where the device will be cemented, the quality of the cementing agent, and the way this agent is selected are points that need to be considered for achieving optimal efficiency in the treatment with orthodontic brackets (4).

Given that moisture can occur during the cementing process for the application of brackets, one must understand its interference mode and relationship with the different adhesive systems available today (5). That said, the aim of this *in vitro* study was to assess the immediate bond strength of metallic brackets cemented to dental enamel subjected to shear bond strength testing.

## Material and Methods

Forty human premolars were selected, analyzed under a stereomicroscope (Stemi DV4, Zeiss Universal Microscope, Jena, Germany), with no cracks or visible structural defects.

**Figure 1:**
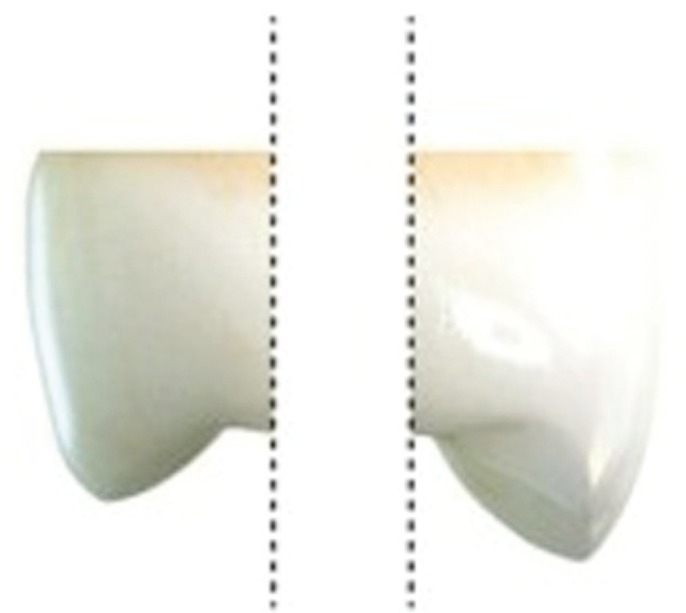
Sketch of a longitudinal section at the coronal portion.

The teeth were sectioned at the cementoenamel junction using a refrigerated diamond disc cutting machine (KG Sorensen, Barueri, SP, Brazil), followed by a section of the coronal portion at the buccal and palatal planes (Fig. 1).

The specimens were placed in cylindrical polyethylene containers measuring 25mm x 21mm and embedded in chemically activated acrylic resin. After polymerization they were polished to ensure flat, smooth and uniform surfaces (Arotec® APL-4, São Paulo, SP, Brazil).

**Figure 2:**
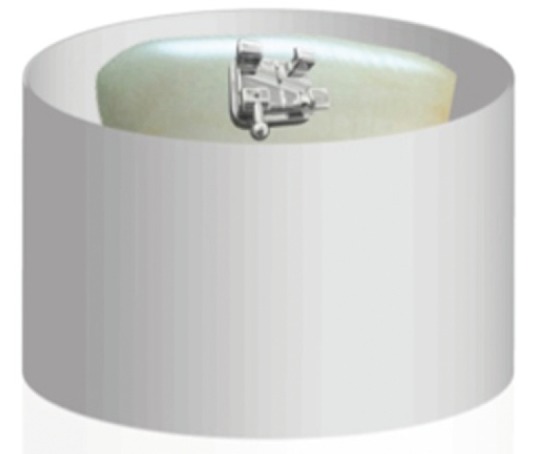
Sketch of an orthodontic bracket cementation.

In the flat area of each specimen, standard edgewise orthodontic brackets were cemented (Agile model, 3M Abzil, Sumaré, SP, Brazil) with a base 1.9 mm high x 3.2 mm wide, totaling an area of 6.08 mm2 (Fig. 2).

The specimens were randomly divided into 10 groups (n=8), differentiated according to their contamination, adhesive system and cementing agent: G1 (control) - no contamination, adhesive system: Transbond™ Plus Self-Etching primer and Transbond™ Color-Changing cement; G2 - contamination prior to application of the adhesive bonding: Transbond™ Plus Self-Etching primer and Transbond™ Color-Changing cement; G3 - contamination after applying the adhesive bonding Transbond™ Plus Self-Etching Primer and Transbond ™ Color-Changing cement; G4 (control) - no contamination, adhesive bonding Transbond™ Plus Self-Etching Primer and Transbond™ XT Light cement; G5 - contamination after applying the adhesive bonding Transbond ™ Plus Self Etching Primer and cement Transbond ™ XT Light; G6 (control) - no contamination, adhesive bonding Transbond™ XT Light Cure Adhesive and Transbond™ XT Light cement; G7 - contamination prior to the application of the adhesive bonding Transbond™ XT Light Cure Adhesive and Transbond™ XT Light cement; G8 - contamination after applying the adhesive bonding Transbond™ XT Light Cure Adhesive and Transbond™ Color-Changing cement; G9 (control) - no contamination, adhesive bonding Transbond™ XT Light Cure Adhesive and Transbond™ Color-Changing cement; G10 - contamination after applying the adhesive bonding Transbond™ XT Light Cure Adhesive and Transbond™ Color-Changing cement.

Prophylaxis was performed with pumice, rinse and air spray for 20 seconds on the groups that received the application of Trans-bond™ Plus Self-Etching Primer (3M Unitek, Monrovia, CA, USA). The adhesive bonding was applied using a disposable micro applicator, and light cured using a LED device (Led SdiRadii Cal, Sdi, Victoria, Australia), with an intensity of 400 mW/cm2 for 20 seconds in the incisal third and 20 seconds in the cervical third. Those that received Transbond™ XT Light Cure adhesive bonding (3M Unitek, Montovia, CA, USA) were cleaned with pumice and were rinsed and dried thoroughly as described above. After the application of 35% phosphoric acid (Ultra-etch®, Ultradent, South Jordan, UT, USA) for 30 seconds, the adhesive bonding was applied on the tooth surface using a micro disposable applicator, which was photopolymerized as in the previous group. For contamination groups, artificial saliva was used consisting of carmellose sodium, sorbitol, sodium chloride, potassium chloride, calcium chloride dihydrate, magnesium chloride hexahydrate, potassium acid phosphate, methylparaben, and water. Contamination of the teeth was simulated by 3 ml artificial saliva applied to the enamel surface using a micro disposable applicator for 5 seconds, allowing for the full surface contamination. Adhesive bonding or cement was used following the protocol of the experimental group, with no drying step.

**Figure 3:**
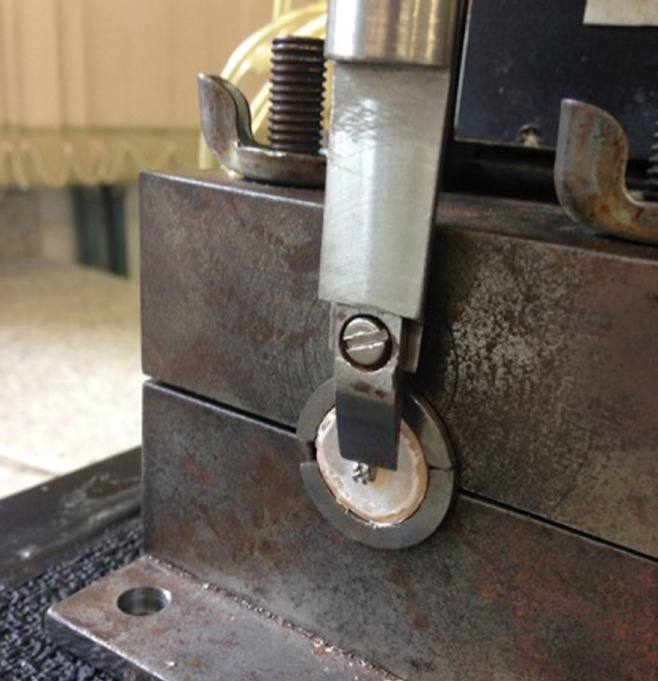
Specimen adapted to a shear-testing device.

The specimens were adapted to a universal testing machine (Instron Universal 4440- C6600, Barueri, SP, Brazil) with the base of the bracket fins perpendicular to the applied force (Fig. 3). Then, a shear strength of 1.0 mm/min was applied until the fracture, converting the recorded scores in Newton (N) into MegaPascal (MPa), as follows: MPa = N/area.

All specimens subjected to mechanical shear testing were prepared for the Adhesive Remnant Index (ARI) analysis under a stereoscopic microscope (Stemi DV4, Zeiss Universal Microscope, Jena, Germany) and optical microscope (N107, Coleman, Santo André, SP, Brazil) at 40X magnification. The following items were adopted as evaluation criteria: (0) no adhesive left on the enamel; (1) less than half of the adhesive left on the enamel; (2) more than half of the adhesive left on the enamel; (3) all the cement left on the enamel.

After data tabulation, two-way ANOVA and Tukey’s test for multiple comparisons were performed, at 95% significance level (*p*<0.05), to determine statistically significant differences.

## Results

The normal distribution of data identified by the Shapiro-Wilk test allowed for the use of a parametric test for the determination of possible significant differences between the groups (*p*=0.05). Analysis of variance identified a statistically significant difference between groups (*p*=0.01), as described in  Table 1.

**Table 1:**
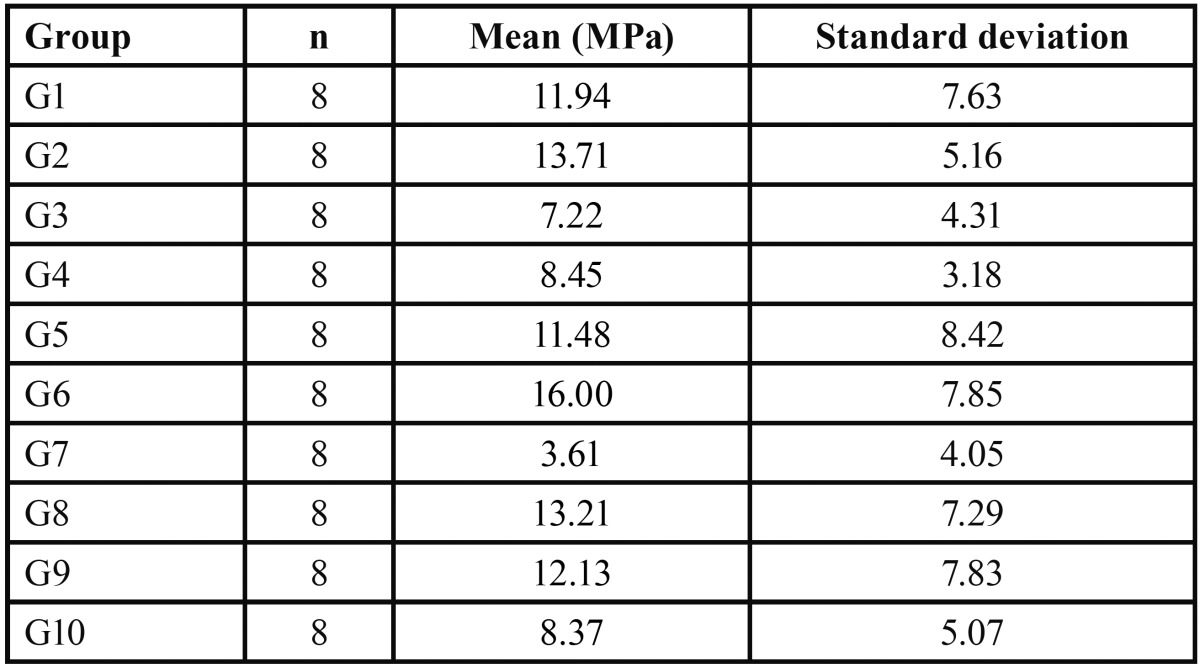
Mechanical shear strength test results for the experimental group.

In order to identify such a difference, the data were subjected to Tukey’s test for multiple comparisons ( Table 2).

**Table 2:**
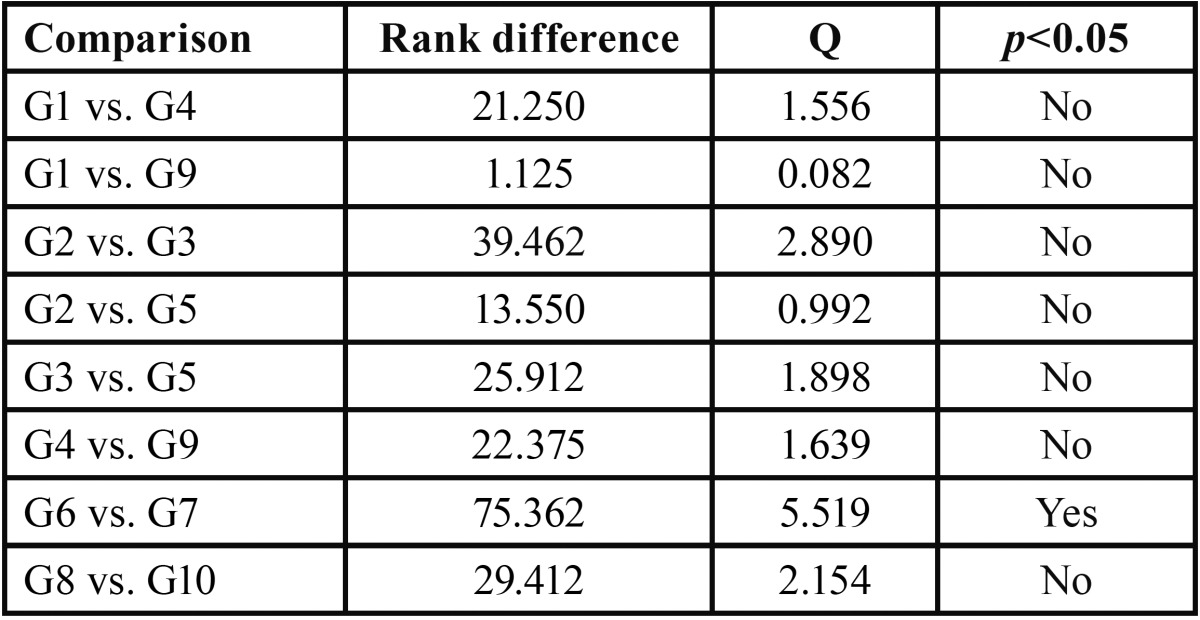
Results of Tukey’s test for multiple comparisons.

ARI analysis under optical microscopy arranged the data in percentages per experimental group ( Table 3).

**Table 3:**
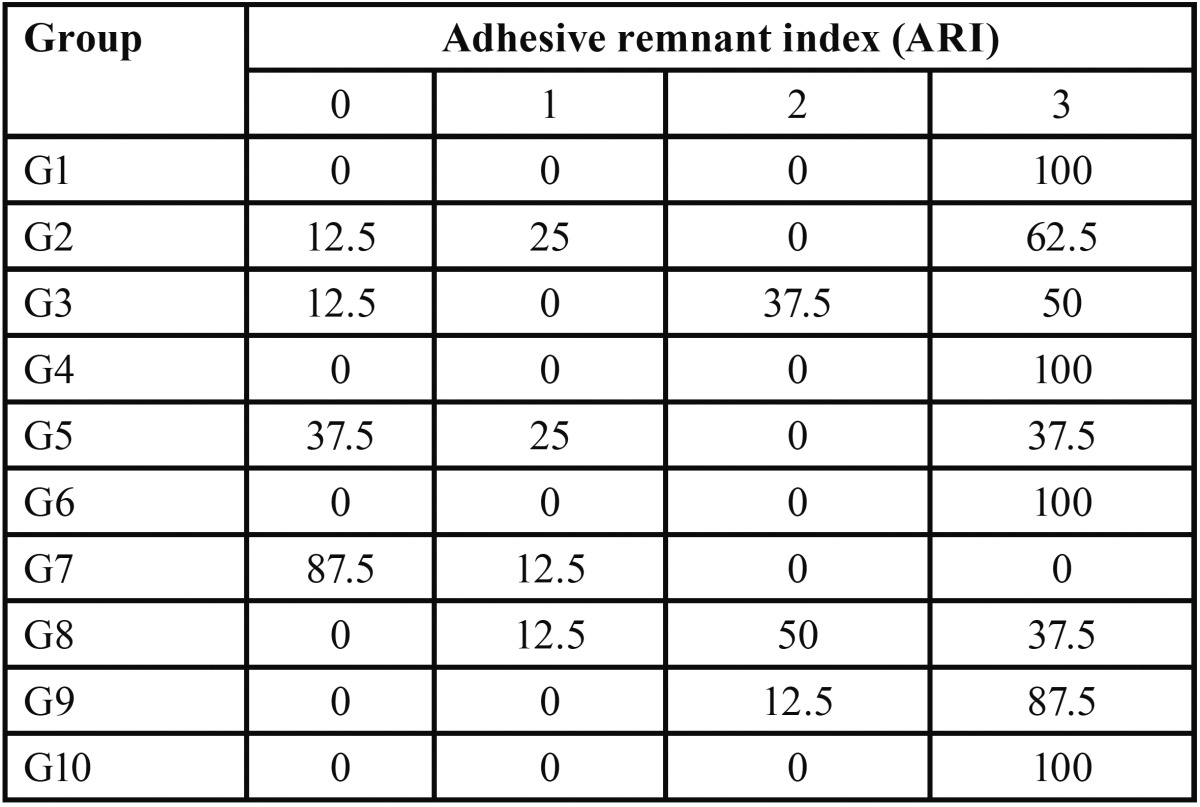
Percentage analysis of Adhesive Remnant Index per experimental group.

## Discussion

The results of ANOVA and Tukey’s statistical tests revealed that saliva contamination showed different behaviors according to the adhesive system and cement used. While most of the groups did not differ statistically from the control group, the specimens conditioned with a simplified conventional system (Transbond™ XT Light Cure Adhesive) cemented with Transbond™ XT Light presented the highest technical sensitivity indices. Thus, the null hypothesis stating that contamination with saliva does not affect the bond strength of orthodontic resin cements was rejected.

The advent of the enamel surface etching technique, initially proposed by Buonocore (6), enabled orthodontics to make a significant advancement, allowing for the direct bonding of orthodontic brackets instead of the dental bandage system. This improvement has provided less aesthetic harm to the patient and a significant reduction in the time required for the system installation (7). In the 1970s, Newman (8) advocated that direct cementation of orthodontic brackets because they allowed for a lower degree of enamel decalcification and less irritation to soft tissues. A number of studies (9,10) have been conducted to measure bond strength required for clinical success. The literature has advocated scores between 5.9 and 7.9 MPa, not considering the action of different tensile stress. Considering the bonding systems and cements used in contamination-free surfaces, the results of this study support the findings of other studies.

Conventional bonding systems are of unquestionable quality and have been supported by their long tradition of good clinical and laboratory results (11) as compared to self-etching systems. This could be seen in the highest scores of uncontaminated groups conditioned with a simplified conventional system (G6 and G9) as compared to the self-etching system (G1 and G4), although no statistically significant difference between them occurred. However, high bond strength is not the sole criterion for a successful bonding (12). Considering that brackets and other orthodontic appliances are removed in about three years, maximum tensile stress is not a vital requirement in orthodontic bonding, but may aggravate the possibility of enamel fracture during bracket de-bonding (13).

Scientific studies have been published since the mid-1980s confronting the clinical results of bonding brackets in environments with and without saliva contamination (14). In this study, the groups that received contamination prior to the application of the adhesive system (G2 and G7) did not show any statistically significant difference as compared to the control group. Nevertheless, the results shown in Table 1 suggest that hydrophilicity of self-etch systems (G2) accounted for the increase in bond strength scores, which were similar to those of the control group (G1), reinforcing the findings by Scougall Vilchis *et al.* (15) and Vincent *et al.* (16). Analyzing the results of the groups contaminated after applying the adhesive bonding (G3, G5, G8 and G10) no statis-tically significant differences were observed between them, although the results presented lower scores than in the control groups (G1, G4, G6 and G9), which supports the findings by Campoy *et al.* (17). Among other reasons, this may be explained by the penetration of saliva proteins and compounds in micro retentions created in the enamel bonding system, compromising its adhesive capacity (18).

The hydrophobic adhesive system presented the highest bond strength scores when applied on a contaminated surface (G7) and was the only one that showed a statistically significant difference as compared to the control group (G6). These findings support those by Prasad *et al.* (19) and Sfrondini *et al.* (20) and have been discussed as being a result of the loss of mechanical retention and decreased surface-free energy. Facing the contamination obstacle, the enamel surface should receive acid etching conditioning again previously to the infiltration of adhesive monomers, being the only effective way to circumvent bonding loss caused by the formation of an organic cuticle and loss of wetting ability (21).

Adhesive remnant index (ARI) results were categorized according to the type of adhesive system used. Hydrophilicity of self-etch adhesive systems had their best use in contaminated environments, in line with findings from previous studies (22). This fact was demonstrated by the higher prevalence of fractures between the bracket and the cement (type 3), showing satisfactory mechanical properties in orthodontic bonding (23), which could be observed in group G7, in which a hydrophobic adhesive applied after saliva contamination presented a high incidence of adhesive failures. Therefore, anticipating the presence of moisture, the use of a hydrophilic primer was able to considerably reduce the chance of bracket debonding. However, such a choice was irrelevant in a contamination-free environment, as it could be seen in the comparison between the groups G1, G4, G6 and G9.

The simplification of *in vitro* methodologies do not provide a perfect reproduction of tensile stress that causes adverse changes in the oral cavity (24). Thus, the results of this study point to the need for long-term assessment of the relationship between contamination, bonding systems, and contemporary orthodontic resin cements.

Despite the limitations of *in vitro* studies, the results obtained in this study allowed us to conclude that saliva contamination did not decrease the immediate bond strength of metal brackets bonded to enamel surfaces, except when the simplified conventional system Transbond XT™ Light cure adhesive was used after saliva contamination.

The use of a conventional hydrophobic bonding system in wet environment accounted for a predominantly adhesive fracture pattern.

## References

[1] Abdelnaby YL, Al-Wakeel Eel S (2010). Effect of early orthodontic force on shear bond strength of orthodontic brackets bonded with different adhesive systems. Am J Orthod Dentofacial Orthop.

[2] Cunha TM, Behrens BA, Nascimento D, Retamoso LB, Lon LF, Tanaka O (2012). Blood contamination effect on shear bond strength of an orthodontic hydrophilic resin. J Appl Oral Sci.

[3] Juneja R, Duhan J, Tewari S, Sangwan P, Bhatnagar N (2014). Effect of blood contamination and decontamination protocols on acetone-based and ethanol-based total etch adhesive systems. J Esthet Restor Dent.

[4] Kano H, Kurogi T, Shimizu T, Nishimura M, Murata H (2012). Viscosity and adhesion strength of cream-type denture adhesives and mouth moisturizers. Dent Mater J.

[5] Bishara SE, Oonsombat C, Ajlouni R, Denehy G (2002). The effect of saliva contamination on shear bond strength of orthodontic brackets when using a self-etch primer. Angle Orthod.

[6] Buonocore MG (1955). A simple method of increasing the adhesion of acrylic filling materials to enamel surfaces. J Dent Res.

[7] Miura F, Nakagawa K, Masuhara E (1971). New direct bonding system for plastic brackets. Am J Orthod.

[8] Newman GV (1971). Clinical treatment with bonded plastic attachments. Am J Orthod.

[9] Urabe H, Rossouw PE, Titley KC, Yamin C (1999). Combinations of etchants, composite resins, and bracket systems: an important choice in orthodontic bonding procedures. Angle Orthod.

[10] Trimpeneers LM, Dermaut LR (1996). A clinical trial comparing the failure rates of two orthodontic bonding systems. Am J Orthod Dentofacial Orthop.

[11] Seemann R, Flury S, Pfefferkorn F, Lussi A, Noack MJ (2014). Restorative dentistry and restorative materials over the next 20 years: a Delphi survey. Dent Mater.

[12] Hori FS, de Carvalho RC (2012). Experimental adhesives with different hydrophilicity: microshear test in after 1, 7, and 90 days' storage. J Adhes Dent.

[13] Bolaños-Carmona V, Zein B, Menéndez-Núñez M, Sánchez-Sánchez P, Ceballos-García L, González-López S (2015). Influence of the bracket on bonding and physical behavior of orthodontic resin cements. Dent Mater J.

[14] Evans LB, Powers JM (1985). Factors affecting in vitro bond strength of no-mix orthodontic cements. Am J Orthod.

[15] Scougall Vilchis RJ, Yamamoto S, Kitai N, Yamamoto K (2009). Shear bond strength of orthodontic brackets bonded with different self-etching adhesives. Am J Orthod Dentofacial Orthop.

[16] Vicente A, Toledano M, Bravo LA, Romeo A, de la Higuera B, Osorio R (2010). Effect of water contamination on the shear bond strength of five orthodontic adhesives. Med Oral Patol Oral Cir Bucal.

[17] Campoy MD, Plasencia E, Vicente A, Bravo LA, Cibrián R (2010). Effect of saliva contamination on bracket failure with a self-etching primer: a prospective controlled clinical trial. Am J Orthod Dentofacial Orthop.

[18] Erickson RL, Barkmeier WW, Latta MA (2009). The role of etching in bonding to enamel: a comparison of self-etching and etch-and-rinse adhesive systems. Dent Mater.

[19] Prasad M, Mohamed S, Nayak K, Shetty SK, Talapaneni AK (2014). Effect of moisture, saliva, and blood contamination on the shear bond strength of brackets bonded with a conventional bonding system and self-etched bonding system. J Nat Sci Biol Med.

[20] Sfondrini MF, Gatti S, Scribante A (2011). Effect of blood contamination on shear bond strength of orthodontic brackets and disinclusion buttons. Br J Oral Maxillofac Surg.

[21] Rotta M, Bresciani P, Moura SK, Grande RH, Hilgert LA, Baratieri LN (2007). Effects of phosphoric acid pretreatment and substitution of bonding resin on bonding effectiveness of self-etching systems to enamel. J Adhes Dent.

[22] Shukla C, Maurya R, Jain U, Gupta A, Garg J (2014). Moisture insensitive primer: A myth or truth. J Orthod Sci.

[23] Delannée M, Grégoire G, Vergnes JN, Sharrock P (2013). Fluid flow through dentin-self-etch resin interface during long term in vitro aging. Mater Sci Eng C Mater Biol Appl.

[24] De Munck J, Vargas M, Van Landuyt K, Hikita K, Lambrechts P, Van Meerbeek B (2004). Bonding of an auto-adhesive luting material to enamel and dentin. Dent Mater.

